# A Pan-Cancer Analysis of Transcriptome and Survival Reveals Prognostic Differentially Expressed LncRNAs and Predicts Novel Drugs for Glioblastoma Multiforme Therapy

**DOI:** 10.3389/fgene.2021.723725

**Published:** 2021-08-24

**Authors:** Rongchuan Zhao, Xiaohan Sa, Nan Ouyang, Hong Zhang, Jiao Yang, Jinlin Pan, Jinhui Gu, Yuanshuai Zhou

**Affiliations:** ^1^Division of Life Sciences and Medicine, School of Biomedical Engineering (Suzhou), University of Science and Technology of China, Heifei, China; ^2^Jiangsu Key Laboratory of Medical Optics, Suzhou Institute of Biomedical Engineering and Technology, Chinese Academy of Sciences, Suzhou, China; ^3^School of Life Sciences, Shanghai University, Shanghai, China; ^4^Department of Anorectum, Suzhou Hospital of Traditional Chinese Medicine, Suzhou, China

**Keywords:** pan-cancer, long noncoding RNA, prognosis, biomarker, glioblastoma multiforme

## Abstract

Numerous studies have identified various prognostic long non-coding RNAs (LncRNAs) in a specific cancer type, but a comprehensive pan-cancer analysis for prediction of LncRNAs that may serve as prognostic biomarkers is of great significance to be performed. Glioblastoma multiforme (GBM) is the most common and aggressive malignant adult primary brain tumor. There is an urgent need to identify novel therapies for GBM due to its poor prognosis and universal recurrence. Using available LncRNA expression data of 12 cancer types and survival data of 30 cancer types from online databases, we identified 48 differentially expressed LncRNAs in cancers as potential pan-cancer prognostic biomarkers. Two candidate LncRNAs were selected for validation in GBM. By the expression detection in GBM cell lines and survival analysis in GBM patients, we demonstrated the reliability of the list of pan-cancer prognostic LncRNAs obtained above. By constructing LncRNA-mRNA-drug network in GBM, we predicted novel drug-target interactions for GBM correlated LncRNA. This analysis has revealed common prognostic LncRNAs among cancers, which may provide insights into cancer pathogenesis and novel drug target in GBM.

## Introduction

Non-coding RNAs (ncRNAs), including microRNA (miRNA), circRNA, long non-coding RNA (LncRNA), and many other kind of RNAs, are non-protein coding transcripts, which had been regarded as useless molecules, accounting for more than 95% of human genome (Tao et al., 2015). However, accumulating evidence indicates that ncRNAs have multiple functions in physiological and pathological processes, including cell growth, proliferation and apoptosis (Penna et al., 2015).

LncRNAs are a novel class of ncRNAs that are longer than 200 nucleotides, with no protein-coding capability (Ulitsky and Bartel, 2013). It has been shown that LncRNAs can elicit gene activation or suppression by interacting with proteins, DNAs and RNAs including miRNAs (Kataoka and Wang, 2014). They can also act as molecular signals, decoys, guides, and scaffolds for transcription factors and epigenetic modifiers (Wang and Chang, 2011).

A number of studies have revealed that LncRNAs are dysregulated in many cancer types. Several common LncRNAs have been investigated in cancers and the results revealed that they can function as potential biomarkers associated with tumor initiation, progression, and prognosis. For example, neuroblastoma associated transcript 1 (*NBAT1*) is demonstrated as a tumor-suppressing LncRNA and habitually downregulated in several cancers including neuroblastoma, osteosarcoma, ovarian cancer, and breast cancer. Loss of *NBAT1* induces tumor cell proliferation, differentiation, migration, and invasion through interaction with EZH2 and miR-21, or targeting ERK1/2- and AKT-mediated signaling pathway (Pandey et al., 2014; Hu et al., 2015; Yan et al., 2017; Yang et al., 2017). NBAT1 can also inhibit autophagy by suppressing the transcription of ATG7 in non-small cell lung cancer (Zheng et al., 2018). Colon cancer-associated transcript-1 (*CCAT1*) is found to be consistently elevated in multiple types of cancer and plays a critical role in various biological processes such as proliferation, invasion, migration, drug resistance, and survival (Nissan et al., 2012; He et al., 2014; Kim et al., 2014; Deng et al., 2015; Wang et al., 2019b). *CCAT1* has been demonstrated to enhance the expression of c-Myc (Xiang et al., 2014; Younger and Rinn, 2014). *CCAT1* can also stimulate EGFR expression, thereby activating MEK/ERK1/2 and PI3K/AKT signaling pathways (Jiang et al., 2018). Metastasis-associated lung adenocarcinoma transcript 1 (*MALAT1*) plays an important role in the pathogenesis and development of various cancers (Amodio et al., 2018; Liu et al., 2018; Zhao et al., 2018). Previous studies revealed that MALAT1 is upregulated in lung cancer, breast cancer, colorectal cancer, bladder cancer, and hepatocellular carcinoma (Goyal et al., 2021). *MALAT1* epigenetically repress TSC2 transcription *via* recruiting EZH2 to TSC2 promoter regions and thus enhances the apoptosis of cardiomyocytes through autophagy inhibition by regulating TSC2-mTOR signaling (Hu et al., 2019). Although increasing prognostic LncRNAs were identified exclusively in a specific cancer type, a comprehensive pan-cancer analysis is of great significance to be performed for prediction of LncRNAs that may serve as prognostic biomarkers. Identifying new prognostic LncRNA biomarkers is of extreme importance for revealing tumorigenesis underlying mechanisms. Although demonstrating LncRNA’s exact function in cancers is difficult at present, it is possible to evaluate their role in prognosis, which is one of the main goals of cancer research.

Glioma is the most common malignant tumor in central nervous system and accounts for approximately 80% of primary intracranial tumors (Zhou et al., 2013). Based on World Health Organization (WHO) classification, glioma is classified into WHO grade I, II, III, and IV (Agnihotri et al., 2013). Among all types of glioma, glioblastoma multiforme (GBM) is the most aggressive type (a WHO grade IV glioma; Lieberman, 2017; Szopa et al., 2017), with a median survival of 15 months. GBM is characterized by chemoradiotherapy resistance and high risk of recurrence (Abbruzzese et al., 2017; Tian et al., 2019). Temozolomide (TMZ) resistance severely limits the efficacy and has become an important cause of poor prognosis. The 5-year recurrence for GBM is nearly universal. Therefore, there is an urgent need to identify novel therapies for GBM (Clarke et al., 2013; Szopa et al., 2017; Tan et al., 2018).

In our study, in order to get more rigorous analysis, we integrated both TANRIC database and ENCORI database, performed a step-by-step filtering and identified a list of 48 pan-cancer prognostic LncRNAs. Through LncRNA expression detection by qPCR and survival analysis on database in GBM, the reliability of our findings is confirmed. Previous pan-cancer analysis commonly aims to discover novel biomarkers across boundaries between tumor types (Weinstein et al., 2013). We took more concentrations on GBM, because there is few LncRNA targeted drugs for GBM therapy. We constructed an LncRNA-mRNA-drug interaction network to give advice to further drug-related LncRNA research and provide guideline for targeted therapeutics.

## Materials and Methods

### Cell Lines

HUVEC, U87MG, and U251MG GBM cell lines were cultured in DMEM (Lot No. 8119284) supplemented with 10% fetal bovine serum (FBS, Lot No. 42G2095K) at 37°C in a humidified air atmosphere containing 5% CO_2_. GSC23 GBM stem cell line was cultured in DMEM F-12 (Lot No. RNBG2219) supplemented with EGF (20 ng/ml, Lot No. PHG0311), bFGF (20 ng/ml, Lot No. PHG0368), B27 (1×, Lot No. 17504044), and NEAA (1×, Lot No. 11140050) at 37°C in a humidified air atmosphere containing 5% CO_2_. DMEM F-12 was purchased from SIGMA, other reagents were purchased from Gibco.

### Data Collection and Preprocessing

Gene expression data (LncRNA sequencing profiles) and corresponding clinical data of 12 cancer types were obtained from the Atlas of ncRNA in Cancer (TANRIC) database based on The Cancer Genome Atlas Data (TCGA) and Cancer Cell Line Encyclopedia (CCLE).^1^ We focused on 12 types of cancers, each with more than 10 normal control samples, including bladder urothelial carcinoma (BLCA, 252 tumor samples and 19 normal samples), breast invasive carcinoma (BRCA, 837 tumor samples and 105 normal samples), head and neck squamous cell carcinoma (HSNC, 426 tumor samples and 42 normal samples), kidney chromophobe (KICH, 66 tumor samples and 25 normal samples), kidney renal clear cell carcinoma (KIRC, 448 tumor samples and 67 normal samples), kidney renal papillary cell carcinoma (KIRP, 198 tumor samples and 30 normal samples), liver hepatocellular carcinoma (LIHC, 200 tumor samples and 50 normal samples), lung adenocarcinoma (LUAD, 488 tumor samples and 58 normal samples), lung squamous cell carcinoma (LUSC, 220 tumor samples and 17 normal samples), prostate adenocarcinoma (PRAD, 374 tumor samples and 52 normal samples), stomach adenocarcinoma (STAD, 285 tumor samples and 33 normal samples), and thyroid carcinoma (THCA, 497 tumor samples and 59 normal samples; Table 1). LncRNA ID was annotated according to GENCODE Release 29 (GRCh38.p12).^2^

**Table 1:** LncRNA expression data of 12 cancer types in TANRIC database.

Data source	Cancer type	Normal samples	Tumor samples
TCGA	BLCA	19	252
TCGA	BRCA	105	837
TCGA	HNSC	42	426
TCGA	KICH	25	66
TCGA	KIRC	67	448
TCGA	KIRP	30	198
TCGA	LIHC	50	200
TCGA	LUAD	58	488
TCGA	LUSC	17	220
TCGA	PRAD	52	374
TCGA	STAD	33	285
TCGA	THCA	59	497

### Identification of Differentially Expressed LncRNAs in Pan-Cancer

The differentially expressed LncRNAs (DELncs) between tumor samples and normal samples were identified using DESeq2 package of R software. The value of *p* was adjusted by multiple significant tests with Bonferroni method. |log2 fold change (FC) | > 1 and *p* < 0.05 were set as the cutoff criteria. Hierarchical Cluster analysis was performed according to the expression values of DELncs. The heatmaps and volcano maps were plotted based on ggplot2 package of R software.

### Survival Analysis of Differentially Expressed LncRNAs in Pan-Cancer

The survival data of the 30 TCGA cancer types and GBM were obtained from ENCORI Pan-Cancer Analysis Platform (Li et al., 2014) and TANRIC database, respectively. Besides the 12 types of cancers in Table 1, we also downloaded overall survival information of adrenocortical carcinoma (ACC), cervical squamous cell carcinoma and endocervical adenocarcinoma (CESC), cholangiocarcinoma (CHOL), colon adenocarcinoma (COAD), lymphoid neoplasm diffuse large B-cell lymphoma (DLBC), esophageal carcinoma (ESCA), acute myeloid leukemia (LAML), brain lower grade glioma (LGG), mesothelioma (MESO), ovarian serous cystadenocarcinoma (OV), pheochromocytoma and paraganglioma (PCPG), prostate adenocarcinoma (PRAD), rectum adenocarcinoma (READ), sarcoma (SARC), skin cutaneous melanoma (SKCM), testicular germ cell tumors (TGCT), thymoma (THYM), uterine corpus endometrial carcinoma (UCEC). The survival data of GBM was obtained from The Atlas of ncRNA in Cancer (TANRIC) based on TCGA and Cancer Cell Line Encyclopedia (CCLE). Patients were separated into higher and lower risk groups by median LncRNA expression. By Kaplan–Meier survival analysis, LncRNAs with Log-rank *p* < 0.05 were considered to be significantly associated with prognosis of patients.

### Functional Enrichment Analysis of GO Annotation and KEGG Pathways

The Pearson correlation coefficient was used to evaluate co-expression relationship between LncRNA and mRNA. Cluster Profiler v3.8 package of R was used to analyze and visualize functional profiles [Gene Ontology, (GO) annotation and Kyoto Encyclopedia of Genes and Genomes (KEGG) pathway] of the co-expressed genes with DELncs. The GO terms and KEGG pathways with *p* < 0.05 was considered as significantly enriched function terms or pathways.

### Quantitative RT-PCR (qRT-PCR) Analysis

Total RNA from HUVEC, U87MG, U251MG, and GSC23 cell lines was isolated using RNAiso Plus (TaKaRa, code: 9109). RNA was transcribed to cDNA using PrimeScript™ RT Reagent Kit with gDNA Eraser (TaKaRa) following the manufacturer’s instructions. Real-time quantitative PCR (qPCR) was performed using SYBR Green Real-time PCR Master Mix (TOYOBO, Lot No.857300) with primers against selected LncRNAs (primer sequences are listed in Table 2). Amplification and real time measurement of PCR products was performed with QuantStudio Real-Time PCR System (Thermo Fisher Scientific). The comparative Ct method was used to quantify the expression levels of LncRNAs. GAPDH gene expression served as an internal control.

**Table 2:** Primer sequences of LINC0008 and RP11-399O19.9.

LncRNA	Primer
LINC00087	F: 5'-GGCTTGGCGGTTCGGCTGTC-3'
LINC00087	R: 5'-GCACTTGCAGGCGGACGTTGA-3'
RP11-399O19.9	F: 5'-CAGAAGTAGGGCAAGTTAGG-3'
RP11-399O19.9	R: 5'-CTCCACTGTCTTCCTCCC-3'

### Predicting lncRNA-mRNA-Drug Interactions for GBM

In the drug discovery and repositioning process, computational prediction of drug-target interactions (DTIs) plays a key role in identifying putative new drugs or novel targets for existing drugs (Savitski et al., 2014; Chernobrovkin et al., 2015; Franken et al., 2015; Cheng et al., 2016; Guney et al., 2016; Mehmood et al., 2016). Among multiple computational approaches, DTINET is a new computational pipeline, which can integrate heterogeneous information to predict new DTIs and repurpose existing drugs (Luo et al., 2017).

## Results

### The Integrative Pipeline for Identification of Pan-Cancer Prognostic DELncs

**Figure 1:**
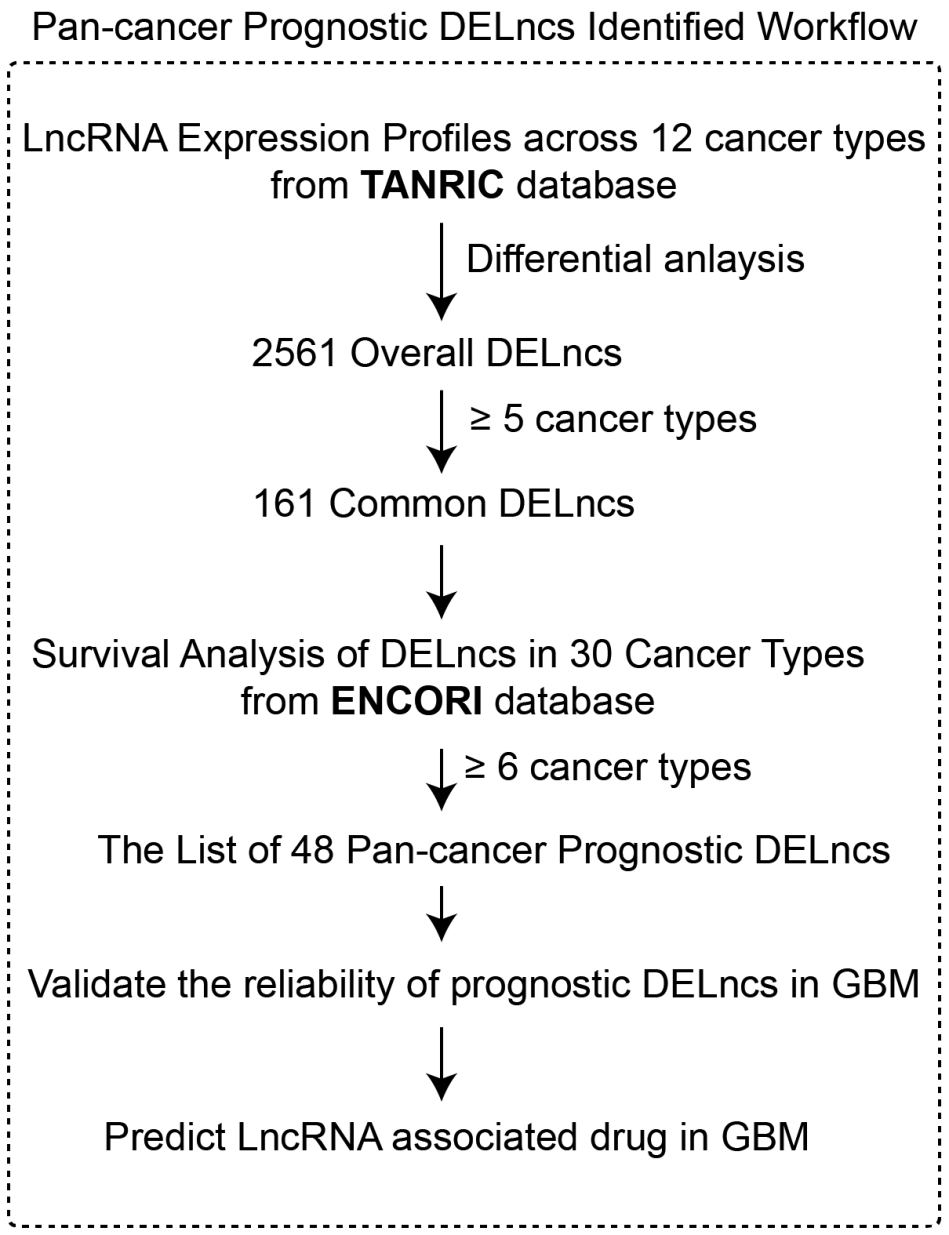
The integrative pipeline for identification of pan-cancer prognostic DELncs.

Figure 1 shows a scheme of the integrative pipeline containing multi-step of data integration and analysis for the identification of pan-cancer prognostic DELncs, together with its validation and application in GBM. First, we performed differential analysis on LncRNA expression profiles in TANRIC database and found 2,561 DELncs across 12 cancer types. Then we identified 161 of these overall DELncs as common DELncs because of their common changing trends in more than five cancer types. Based on the survival information in ENCORI database, we filtered out more than half of common DELncs with Log-rank *p* < 0.05 in less than six cancer types and acquired a list of 48 pan-cancer prognostic DELncs. Afterward, we validate the reliability of our list in GBM using both qPCR and database analysis. Finally, we construct an LncRNA-mRNA-drug network in GBM and predicted potential LncRNA associated drugs in GBM.

### Comparison of Differentially Expressed LncRNAs in Pan-Cancer

**Figure 2:**
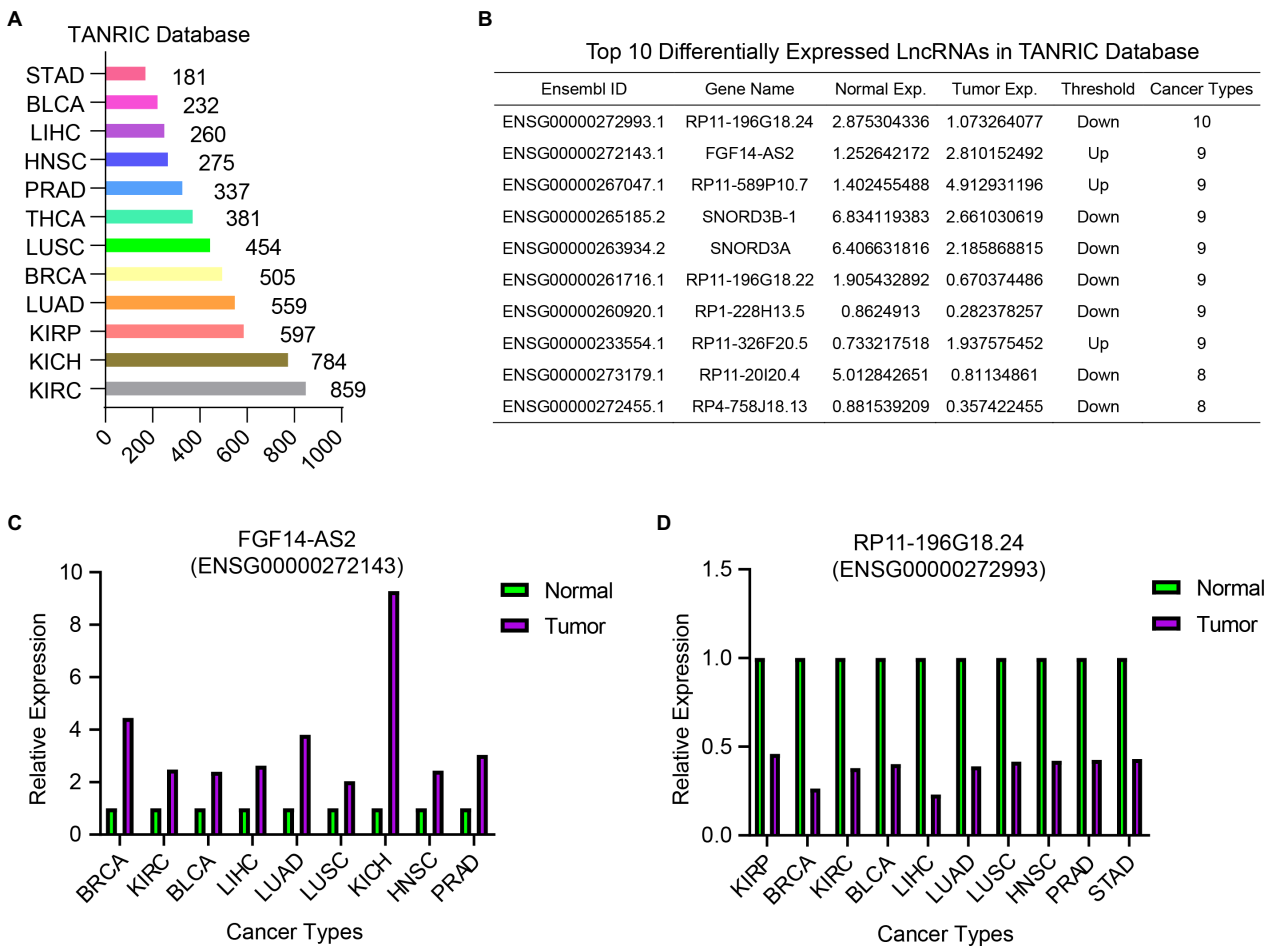
Differentially expressed LncRNAs analysis in 12 TCGA cancer types between tumor samples and normal samples. **(A)** The number of identified DELncs in each cancer type in TANRIC database. **(B)** A list of top 10 common DELncs in 12 cancer types. **(C)** The relative expression of FGF14-AS2 in BRCA, KIRC, BLCA, LIHC, LUAD, LUSC, KICH, HNSC, and PRAD samples compared with normal samples. **(D)** The relative expression of RP11-196G18.24 in KIRP, BRCA, KIRC, BLCA, LIHC, LUAD, LUSC, HNSC, PRAD, and STAD samples compared with normal samples.

To identify common DELncs in different cancer types, we compared the LncRNA expression profiles between tumor samples and paired normal samples in 12 cancer types from TCGA database, including BLCA, BRCA, HSNC, KICH, KIRC, KIRP, LIHC, LUAD, LUSC, PRAD, STAD, and THCA. The result of differential analysis indicated that there were 2,561 DELncs across 12 cancer types altogether, where 859 DELncs were identified in KIRC samples and only 181 DELncs in STAD samples (Figure 2A). Among these 2,561 overall DELncs, we found that most of them showed similar tendency in more than one or two cancer types. Here we showed the top list of 10 most common DELncs (Figure 2B). The most representative up-regulated LncRNA is FGF14-AS2 (Ensembl ID: ENSG00000272143.1) and the most downregulated LncRNA is RP11-196G18.24 (Ensembl ID: ENSG00000272993.1). FGF14-AS2 was consistently upregulated in nine cancer types, including BRCA, KIRC, BLCA, LIHC, LUAD, LUSC, KICH, HNSC, and PRAD (Figure 2C). RP11-196G18.24 showed downregulation in almost all the cancer types except KIRC and THCA (Figure 2D).

### Survival Analysis and Functional Annotation of DELnc in Pan-Cancer

**Figure 3:**
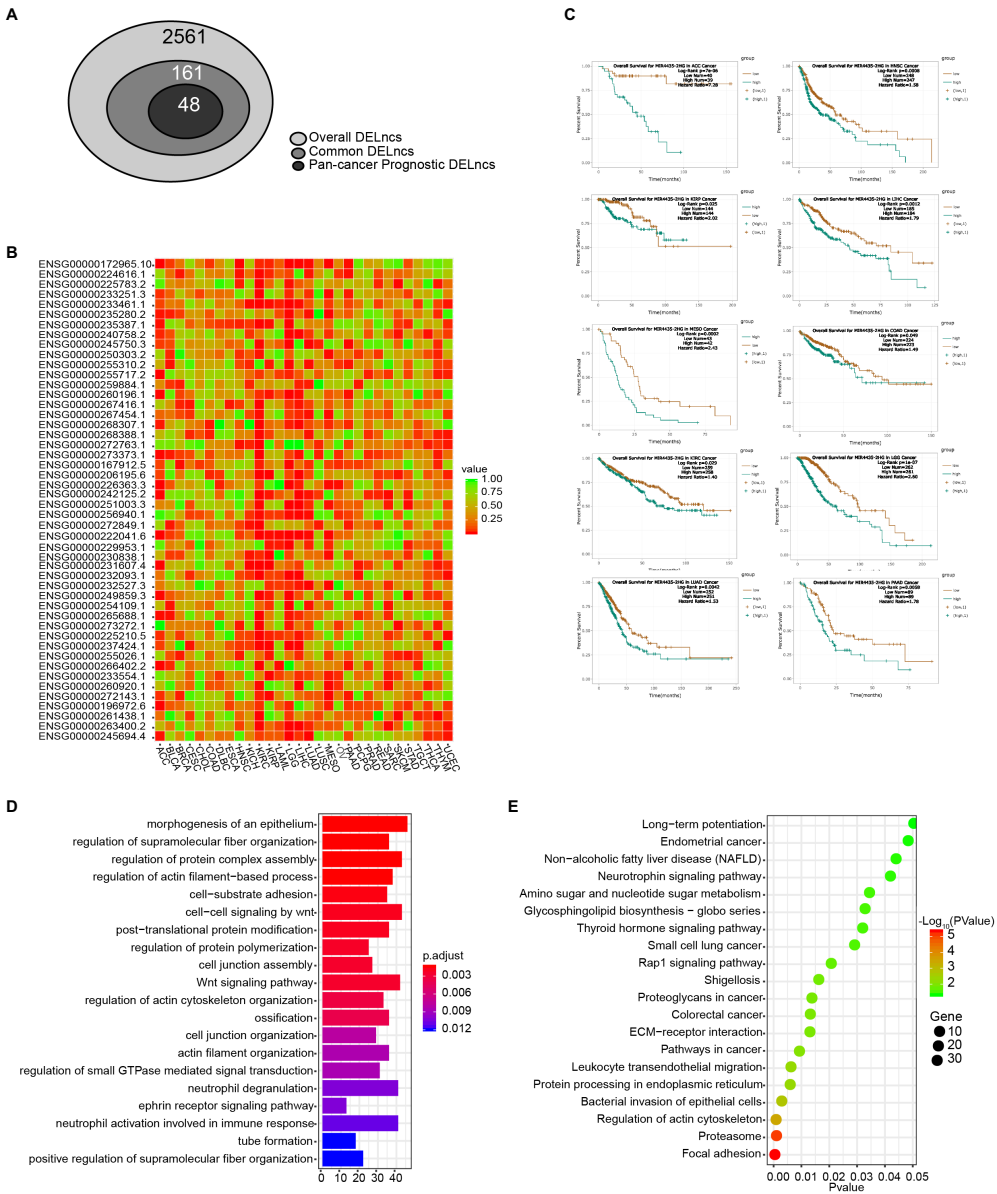
Survival analysis and functional annotation of DELncs. **(A)** Identification of common DELncs and pan-cancer prognostic DELncs. **(B)** Clustering and heatmap of 48 DELncs’ prognostic value by the value of *p* of Log-rank survival analysis in 30 cancer types. **(C)** Kaplan–Meier survival analysis of MIR4435-2HG in ACC, COAD, HNSC, KIRC, KIRP, LGG, LIHC, LUAD, MESO, and PAAD. **(D)** Gene Ontology (GO) annotation of MIR4435-2HG by its correlation mRNA expression. **(E)** Kyoto Encyclopedia of Genes and Genomes (KEGG) pathway enrichment analysis of MIR4435-2HG by its correlation mRNA expression.

In order to acquire those LncRNAs that may serve as potential prognostic biomarkers of pan-cancer, we perform a step-by-step filtering (Figure 3A). In the initial loose screening step, 161 DELncs were selected due to their similar expression trends in more than five among 12 cancer types. Then the survival analysis in 30 cancer types in online tool ENCORI was performed to examine the relationship between 161 LncRNAs and the prognosis of cancer patients. In a more stringent step, 48 DELncs with Log-rank *p* < 0.05 in more than six cancer types were considered to be associated with prognosis of pan-cancer and were selected for further investigation (Figure 3B). In this way, we were able to take more LncRNAs in consideration and acquire a concise list of pan-cancer prognotic DELncs for further research.

Cluster analysis and heatmap were performed according to the value of *p* of Log-rank of the overall survival analysis of these 48 LncRNAs in 30 cancer types (Figure 3B). Among the 48 candidates, the top one LncRNA *MIR4435-2HG* (Ensembl ID: ENSG00000172965.10) was significantly associated with the 10 cancer types (Log-rank p value < 0.05), including ACC, COAD, HNSC, KIRC, KIRP, LGG, LIHC, LUAD, MESO, and PAAD. Kaplan–Meier survival estimate in ENCORI Pan-Cancer Analysis Platform revealed that higher expression of *MIR4435-2HG* in 10 cancer types was robustly associated with worse prognosis (Figure 3C). In order to uncover the biological functions of *MIR4435-2HG*, we performed the GO annotation and KEGG pathway enrichment analysis. As shown in Figures 3D,E, the *MIR4435-2HG* co-expressed genes were associated with the category of morphogenesis of an epithelium, regulation of protein complex assembly, Wnt signaling pathway, and cell-substrate adhesion (Figure 3D). KEGG pathway enrichment analysis revealed that the genes associated with *MIR4435-2HG* were mainly enriched in focal adhesion, leukocyte trans-endothelial migration, and pathway in cancer (Figure 3E).

### Evaluation of DELncs’ Expression and Prognostic Value in GBM

To further validate the expression of 48 DELncs from Pan-cancer analysis, we selected two (RP11-399O19.9 and LINC00087) of them that have not been well studied to assess the reliability of differentially expression analysis in pan-cancer. Here, we chose GBM as our validation set. GBM is highly aggressive grade 4 glioma and is the most common type of malignant glioma, with 10,000 new diagnoses each year. However, there were few LncRNA revealed to be associated with the diagnosis and prognosis of GBM.

**Figure 4:**
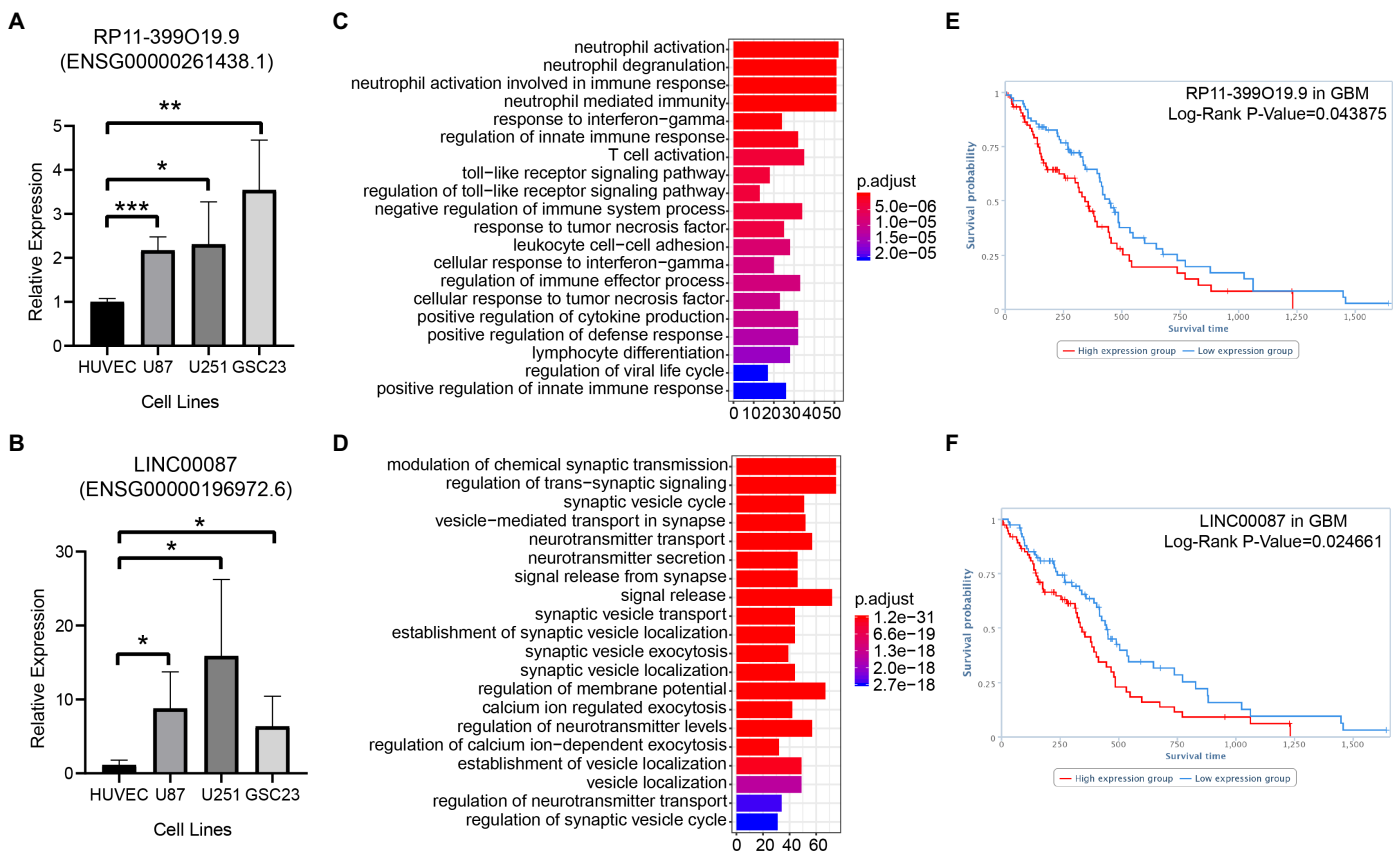
Assess the reliability of candidate LncRNAs by the examination of RP11-399O19.9 and LINC00087 in GBM. **(A)** mRNA expression of RP11-399O19.9 and **(B)** LINC00087 in HUVEC, U87MG, U251MG, and GSC23 cell lines (*means *p* < 0.05, **means *p* < 0.01, and ***means *p* < 0.001). **(C)** GO annotation of RP11-399O19.9 and **(D)** LINC00087. **(E)** Kaplan–Meier survival analysis of RP11-399O19.9 and **(F)** LINC00087 in GBM patients.

The relative expression of RP11-399O19.9 (Ensembl ID: ENSG00000261438.1) and LINC00087 (Ensembl ID: ENSG00000196972.6) in U87MG, U251MG, and GSC23 was up regulated compared with normal cell line HUVEC (Figures 4A,B). GO annotation was performed to predict the potential biological processes of RP11-399O19.9 and LINC00087. Based on TANRIC database, RP11-399O19.9 co-expressed genes were correlated with the categorical terms of neutrophil activation, neutrophil degranulation, and neutrophil activation involved in immune response and many other processes of immune system (Figure 4C). This indicated that RP11-399O19.9 may play an important role in the regulation of immune system, especially neutrophil activation. Genes associated with LINC00087 were enriched in the modulation of chemical synaptic transmission, the regulation of trans-synaptic signaling, and synaptic vesicle cycle (Figure 4D), indicating that LINC00087 might be involved in intercellular signal transmission.

Finally, to investigate the prognostic significance of RP11-399O19.9 and LINC00087 expression in GBM patients, we obtained the overall survival information from TANRIC database. The Kaplan–Meier survival analysis showed that RP11-399O19.9 and LINC00087 are able to separate patients into higher and lower risk groups by median PI, with the value of *p* of Log-rank of 0.043875 and 0.024661 for GBM. High expression of both RP11-399O19.9 and LINC00087 are significantly associated with poor prognosis for GBM patients (Log-rank *p* < 0.05, Figures 4E,F).

### Prediction of LncRNAs as Novel Targets for Existing Drugs in GBM Therapy

**Figure 5:**
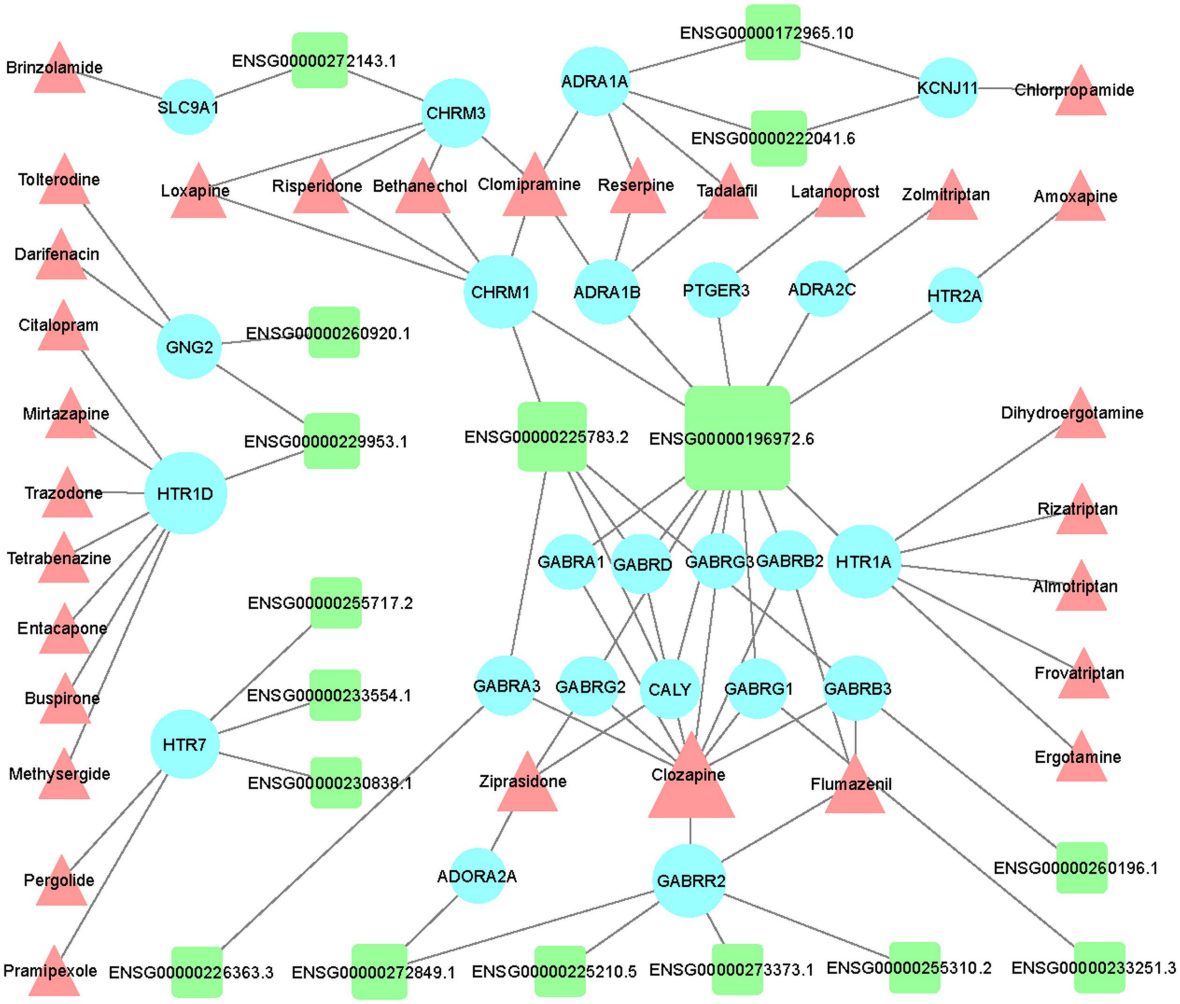
Network visualization of the LncRNA-mRNA-drug interactions in GBM. Visualization of interaction network between differentially expressed LncRNAs and top 150 drug-target interactions (DTIs) predicted by DTINET. LncRNAs, targets (mRNAs), and drugs are shown in green round rectangles, blue circles and pink triangles, respectively.

Based on the list of top 150 novel drug-target interactions (Supplementary Table 1) predicted by DTINet, we constructed an LncRNA-mRNA-drug network to identify the GBM correlated drugs. GBM related LncRNA-mRNA-drug network was visualized using Cytoscape software (Version 3.7.1; Figure 5). In the network, LINC00087 (Ensembl ID: ENSG00000196972.6) has the most interactions with a large amount of drug targets (mRNA), showing the most relevant with existing drugs including Clozapine, Zolmitriptan, Bethanechol, etc. We find several Extrasynaptic γ-aminobutyric acid type A (GABAA) receptors family (GABR) targets, which have interaction with LINC00087 including GABRA1, GABRB2, GABRD, GABRG1,GABRG2, and GABRG3. GABAA receptor family contributes to memory performance. Dysregulation of GABAA receptor expression, which occurs in some neurological disorders, is associated with memory impairment (Whissell et al., 2016). Their related drug, Clozapine (CZP), a dibenzodiazepine atypical antipsychotic drug, was introduced for treatment of schizophrenia in Europe in 1971, rapidly gaining popularity due to its efficacy and virtual absence of extrapyramidal side effects (Mijovic and MacCabe, 2020). Clozapine may be a potential drug for GBM treatment.

## Discussion

Evaluating prognostic value of factors associated with tumorigenesis and progression is an important part of cancer research. Numerous studies have demonstrated that many factors have implication in tumor progression or clinical prognosis in pan-cancer including gene expression, DNA methylation, mutation, etc. Although several LncRNAs have been identified as diagnostic or prognostic markers (Prensner et al., 2011; Sun et al., 2013), a pan-cancer analysis of prognostic LncRNA has rarely been performed. At the same time, there are variations across different cancers in terms of prognosis related LncRNAs, which leads to inconvenience in its utility in clinical oncology. In this study, we analyzed LncRNA expression profiles of 4,848 samples from 12 TCGA cancer types in TANRIC database. We systematically analyzed DELncs between tumor and normal samples in each cancer type and found 2,561 LncRNAs that were simultaneously dysregulated in 12 cancer types. Afterward, we evaluated the prognostic effect of 161 LncRNAs in 30 cancer type and ultimately identified 48 DELncs as our pan-cancer prognostic LncRNAs. *MIR4435-2HG*, as one of the 48 DELncs, showed prognostic importance in 10 cancer types. Previous studies have demonstrated that upregulation of *MIR4435-2HG* is associated with bad prognosis of patients with prostate carcinoma (Zhang et al., 2019), breast cancer (Deng et al., 2016), gastric cancer (Wang et al., 2019a), lung cancer (Qian et al., 2018), and colorectal cancer (Ouyang et al., 2019). Consistent with these studies, the functional annotation of *MIR4435-2HG* in our study indicates that it may play a leading role in cancer cell metastasis and invasion and thus leads to bad prognosis of patients. Although many other LncRNAs in our list have not been demonstrated to have association with tumorigenesis, the analysis we performed above is helpful to predict LncRNAs as cancer markers and may provide directions in cancer research.

Evaluating gene expression in cancer cell lines and association with patients’ prognosis are common methods in cancer research. We did not perform differential expression analysis of LncRNAs in GBM in the first part of results because of lacking LncRNA expression of normal samples of GBM in TANRIC database. We also wanted to acquire common differentially expressed LncRNAs that can give advice to multiple cancer therapies and drug discoveries through pan-cancer analysis. By selecting two of DELncs, detecting their expression in GBM cell lines and analyzing prognosis of GBM patients, we were able to validate the reliability of our 48-DELncs-list. RP11-399O19.9 and LINC00087 have not been well studied, but their dysregulated expressions and prognostic values intimate their importance in tumorigenesis and prognosis. Even if lacking LncRNA transcriptome profile of normal samples of GBM in TCGA database, this study provides a novel method of LncRNA research in GBM.

GBM is considered as incurable intracranial malignant tumor, with a median survival of 15 months following aggressive combination of therapies including maximal-safe surgical resection, adjuvant radiation therapy (RT) with concurrent, and adjuvant temozolomide (TMZ) treatment (Stupp et al., 2009). However, TMZ resistance severely limits the efficacy and has become an important cause of poor prognosis. As TMZ is the only chemotherapy drug available for GBM, it is urgent to look for new drugs or repurpose existing drugs for GBM. Several previous studies indicate that LncRNA may play an important role in GBM. *HOTAIR* could promote glioblastoma cell cycle progression (Zhang et al., 2015); *FOXM1-AS* could enhance self-renewal and tumorigenesis of glioblastoma stem-like cells (Zhang et al., 2017); *H19* could promote glioblastoma cell invasion, angiogenesis, and tube formation (Jia et al., 2016); *MALAT1* could decrease the sensitivity of glioblastoma cells to TMZ (Li et al., 2017). Althogh many LncRNAs have been identified as biomarkers of GBM, there is few LncRNA targeted drugs for GBM therapy. The future of LncRNA-based drug discovery is bright. However, it is still an emerging concept and strategy compared with the traditional drug targets and proteins (Chen et al., 2021). Target selection is a key element of drug development; therefore, identifying the most potential LncRNAs is the first step and the most important process. Further advances in LncRNA-targeted drugs are clearly dependent on the in-depth basic research into the function and mechanisms of LncRNAs. Our study provided a list of 48 LncRNAs by differential expression analysis and survival analysis in pan cancer, which will give advice to the selection of the most potential LncRNAs for further in-depth basic research and LncRNA-based drug discovery. Computational prediction of drug-target interactions (DTIs) is a useful tool for researchers to identify new drugs or novel targets for existing drugs. According to prognostic LncRNA candidates identified, we built LncRNA-mRNA-drug interaction network, which may be beneficial in the treatment of GBM. LINC00087 and Clozapine might be the most valuable LncRNA target and drug in our network for GBM therapy, respectively. In addition, Clomipramine is one of the most widely used tricyclic antidepressants in Western Europe (Balant-Gorgia et al., 1991). Flumazenil appears to act at CNS. It is an antagonist synthesized to competitively block the effects of benzodiazepines on GABAergic pathway-mediated inhibition in the CNS (Votey et al., 1991). Ziprasidone is a recently approved atypical antipsychotic agent (available in oral and short-acting intramuscular formulations) effective in the treatment of schizophrenia in an outpatient setting and in the treatment of acute psychotic episodes (Beedham et al., 2003). These drugs that have been proved to be effective in the treatment of CNS diseases may be effective in GBM treatment.

Since 2012, multiple efforts have launched toward TCGA pan-cancer analysis across many different tumor types (Han et al., 2014; Ching et al., 2016; Luo et al., 2019; Cui et al., 2020). They mainly focused on the mutational landscape (Kandoth et al., 2013). The aim of TCGA pan-cancer initiative is to discover novel intervention strategies, such as discovering novel biomarkers among different tumor samples (Weinstein et al., 2013; Danaher et al., 2018; Gobin et al., 2019). These studies did not make efforts to the LncRNA-based drug discovery. Our research integrated pan cancer analysis with the computational prediction of drug-target interactions together to get 48 DELnc list and its related drugs, which will be of value to both prognostic comments and drug discovery. In our study, we took both LncRNA expression level and prognostic value in consideration and identified a list of 48 pan-cancer prognostic LncRNAs by referring to previous studies. To ensure the reliability of our findings, we validated it in GBM in two aspects: expression level detection by QPCR and survival analysis based on database. We identified these LncRNA not only as biomarkers of pan-cancer but also as novel targets of existing drugs because of their interaction with mRNAs.

In summary, this study provided a list of 48 LncRNAs by differential expression analysis and survival analysis, together with the LncRNA-mRNA-drug interaction network in GBM. The findings also highlighted the prognostic value of LncRNA in pan-cancer research and provided a new perspective for GBM drug target identification. Although we have identified many potential prognostic LncRNAs in multiple cancer types, further research is needed for the evaluation of their function in cancers. Despite limitations of current work, it is a good way to integrate clinical information into LncRNA research in pan-cancer to seek for potential LncRNA targets of cancer therapy and further studies.

## Data Availability Statement

The original contributions presented in the study are included in the article/Supplementary Material, further inquiries can be directed to the corresponding authors.

## Author Contributions

RZ: conceptualization, methodology, formal analysis, writing – original draft, and visualization. XS: formal analysis and data curation. NO and HZ: visualization. JY and JP: writing – review and editing. JG: writing – review and editing and supervision. YZ: writing – review and editing, supervision, and funding acquisition. All authors contributed to the article and approved the submitted version.

## Conflict of Interest

The authors declare that the research was conducted in the absence of any commercial or financial relationships that could be construed as a potential conflict of interest.

## Publisher’s Note

All claims expressed in this article are solely those of the authors and do not necessarily represent those of their affiliated organizations, or those of the publisher, the editors and the reviewers. Any product that may be evaluated in this article, or claim that may be made by its manufacturer, is not guaranteed or endorsed by the publisher.

## Footnotes

^1^ https://ibl.mdanderson.org/tanric/_design/basic/main.html

^2^ https://www.gencodegenes.org/human/
