# Supplementary material for: A Pan-Cancer Analysis of Transcriptome and Survival Reveals Prognostic Differentially Expressed LncRNAs and Predicts Novel Drugs for Glioblastoma Multiforme Therapy
Source: Front Genet. 2021 Aug 24;12:723725. doi: 10.3389/fgene.2021.723725 (PMC8575119; doi:10.3389/fgene.2021.723725)
Supplement: Supplementary file 1 [file Table_1.docx]

Supplementary Table 1: The list of top 150 novel drug-target interactions predicted by DTINet

| Drug ID | Drug Name | Protein ID | Gene Name | Score |
| --- | --- | --- | --- | --- |
| DB00363 | Clozapine | P24046 | GABRR1 | 0.761 |
| DB00363 | Clozapine | P14867 | GABRA1 | 0.75694 |
| DB00363 | Clozapine | P28472 | GABRB3 | 0.75411 |
| DB00363 | Clozapine | P28476 | GABRR2 | 0.75038 |
| DB00363 | Clozapine | P18505 | GABRB1 | 0.74735 |
| DB00363 | Clozapine | P47870 | GABRB2 | 0.72981 |
| DB00246 | Ziprasidone | P41595 | HTR2B | 0.72166 |
| DB00363 | Clozapine | Q99928 | GABRG3 | 0.71139 |
| DB00363 | Clozapine | Q8N1C3 | GABRG1 | 0.70525 |
| DB00363 | Clozapine | P48169 | GABRA4 | 0.68716 |
| DB00953 | Rizatriptan | P08908 | HTR1A | 0.67743 |
| DB00363 | Clozapine | P34903 | GABRA3 | 0.66402 |
| DB00247 | Methysergide | P28221 | HTR1D | 0.62884 |
| DB00247 | Methysergide | P28222 | HTR1B | 0.62314 |
| DB00363 | Clozapine | O14764 | GABRD | 0.61638 |
| DB00960 | Pindolol | P08913 | ADRA2A | 0.55057 |
| DB00706 | Tamsulosin | P25024 | CXCR1 | 0.54856 |
| DB00521 | Carteolol | P08913 | ADRA2A | 0.54836 |
| DB00966 | Telmisartan | P23219 | PTGS1 | 0.54656 |
| DB00966 | Telmisartan | P35354 | PTGS2 | 0.54636 |
| DB00363 | Clozapine | P18507 | GABRG2 | 0.54057 |
| DB00734 | Risperidone | P11229 | CHRM1 | 0.53979 |
| DB00998 | Frovatriptan | P08908 | HTR1A | 0.53629 |
| DB00734 | Risperidone | P08912 | CHRM5 | 0.52257 |
| DB00734 | Risperidone | P20309 | CHRM3 | 0.51599 |
| DB00373 | Timolol | P08913 | ADRA2A | 0.5115 |
| DB00598 | Labetalol | P08913 | ADRA2A | 0.50653 |
| DB00820 | Tadalafil | P35348 | ADRA1A | 0.50256 |
| DB01595 | Nitrazepam | P23416 | GLRA2 | 0.49251 |
| DB00630 | Alendronate | P35354 | PTGS2 | 0.4916 |
| DB00335 | Atenolol | P08913 | ADRA2A | 0.49021 |
| DB00206 | Reserpine | P35348 | ADRA1A | 0.48914 |
| DB00734 | Risperidone | P08173 | CHRM4 | 0.48805 |
| DB00630 | Alendronate | P23219 | PTGS1 | 0.48597 |
| DB00690 | Flurazepam | P23416 | GLRA2 | 0.48486 |
| DB01162 | Terazosin | P25024 | CXCR1 | 0.48477 |
| DB00246 | Ziprasidone | P25021 | HRH2 | 0.48473 |
| DB00246 | Ziprasidone | P08588 | ADRB1 | 0.47867 |
| DB00187 | Esmolol | P08913 | ADRA2A | 0.47196 |
| DB01064 | Isoprenaline | P08913 | ADRA2A | 0.47073 |
| DB01203 | Nadolol | P08913 | ADRA2A | 0.47031 |
| DB00656 | Trazodone | P28221 | HTR1D | 0.46836 |
| DB00656 | Trazodone | P28222 | HTR1B | 0.46427 |
| DB01242 | Clomipramine | P35348 | ADRA1A | 0.46054 |
| DB00612 | Bisoprolol | P08913 | ADRA2A | 0.46001 |
| DB01242 | Clomipramine | P11229 | CHRM1 | 0.45769 |
| DB00654 | Latanoprost | P34995 | PTGER1 | 0.45248 |
| DB00571 | Propranolol | P08913 | ADRA2A | 0.45204 |
| DB00264 | Metoprolol | P08913 | ADRA2A | 0.45169 |
| DB01194 | Brinzolamide | P19634 | SLC9A1 | 0.44778 |
| DB01242 | Clomipramine | P08912 | CHRM5 | 0.44454 |
| DB01242 | Clomipramine | P20309 | CHRM3 | 0.44031 |
| DB01242 | Clomipramine | P08173 | CHRM4 | 0.43676 |
| DB00575 | Clonidine | P08588 | ADRB1 | 0.43122 |
| DB00231 | Temazepam | P23416 | GLRA2 | 0.43086 |
| DB01186 | Pergolide | P34969 | HTR7 | 0.43083 |
| DB00246 | Ziprasidone | Q9NYX4 | CALY | 0.43008 |
| DB00320 | Dihydroergotamine | P08908 | HTR1A | 0.42877 |
| DB01215 | Estazolam | P23416 | GLRA2 | 0.4275 |
| DB00494 | Entacapone | P08913 | ADRA2A | 0.42727 |
| DB00696 | Ergotamine | P08908 | HTR1A | 0.4247 |
| DB01193 | Acebutolol | P08913 | ADRA2A | 0.42232 |
| DB00195 | Betaxolol | P08913 | ADRA2A | 0.42148 |
| DB00672 | Chlorpropamide | Q14654 | KCNJ11 | 0.41954 |
| DB01236 | Sevoflurane | P21917 | DRD4 | 0.41895 |
| DB00794 | Primidone | P21917 | DRD4 | 0.41816 |
| DB00413 | Pramipexole | P34969 | HTR7 | 0.41769 |
| DB00494 | Entacapone | P08588 | ADRB1 | 0.40592 |
| DB00683 | Midazolam | P23416 | GLRA2 | 0.40231 |
| DB00842 | Oxazepam | P23416 | GLRA2 | 0.40061 |
| DB00656 | Trazodone | P08588 | ADRB1 | 0.39912 |
| DB01136 | Carvedilol | P08913 | ADRA2A | 0.39647 |
| DB00363 | Clozapine | P23416 | GLRA2 | 0.39592 |
| DB00363 | Clozapine | P25021 | HRH2 | 0.39498 |
| DB04844 | Tetrabenazine | P28221 | HTR1D | 0.39283 |
| DB00714 | Apomorphine | P08588 | ADRB1 | 0.38813 |
| DB04844 | Tetrabenazine | P28222 | HTR1B | 0.38666 |
| DB00346 | Alfuzosin | P25024 | CXCR1 | 0.38649 |
| DB00363 | Clozapine | P41595 | HTR2B | 0.38579 |
| DB00475 | Chlordiazepoxide | P23416 | GLRA2 | 0.38531 |
| DB00490 | Buspirone | P28222 | HTR1B | 0.38245 |
| DB01558 | Bromazepam | P23416 | GLRA2 | 0.38224 |
| DB01205 | Flumazenil | P47870 | GABRB2 | 0.38213 |
| DB00490 | Buspirone | P28221 | HTR1D | 0.38178 |
| DB01238 | Aripiprazole | P41595 | HTR2B | 0.38125 |
| DB01205 | Flumazenil | P24046 | GABRR1 | 0.38065 |
| DB01205 | Flumazenil | P18505 | GABRB1 | 0.37661 |
| DB00321 | Amitriptyline | P25024 | CXCR1 | 0.37639 |
| DB00843 | Donepezil | P30926 | CHRNB4 | 0.37604 |
| DB01205 | Flumazenil | P28472 | GABRB3 | 0.37583 |
| DB00988 | Dopamine | P08913 | ADRA2A | 0.37088 |
| DB01189 | Desflurane | P21917 | DRD4 | 0.36894 |
| DB01149 | Nefazodone | P08588 | ADRB1 | 0.36839 |
| DB00988 | Dopamine | P08588 | ADRB1 | 0.36729 |
| DB00968 | Methyldopa | P08588 | ADRB1 | 0.36711 |
| DB00619 | Imatinib | P27986 | PIK3R1 | 0.36579 |
| DB00679 | Thioridazine | Q01959 | SLC6A3 | 0.36381 |
| DB00206 | Reserpine | P35368 | ADRA1B | 0.36251 |
| DB00206 | Reserpine | P25100 | ADRA1D | 0.36244 |
| DB01205 | Flumazenil | P28476 | GABRR2 | 0.3622 |
| DB00404 | Alprazolam | P23416 | GLRA2 | 0.36129 |
| DB00215 | Citalopram | P28221 | HTR1D | 0.35962 |
| DB00186 | Lorazepam | P23416 | GLRA2 | 0.35862 |
| DB00215 | Citalopram | P28222 | HTR1B | 0.35812 |
| DB00543 | Amoxapine | P28223 | HTR2A | 0.35802 |
| DB00458 | Imipramine | P25024 | CXCR1 | 0.35779 |
| DB00897 | Triazolam | P23416 | GLRA2 | 0.3565 |
| DB00228 | Enflurane | P21917 | DRD4 | 0.35514 |
| DB00315 | Zolmitriptan | P18825 | ADRA2C | 0.35489 |
| DB00494 | Entacapone | P28221 | HTR1D | 0.35449 |
| DB00472 | Fluoxetine | P30531 | SLC6A1 | 0.35429 |
| DB00363 | Clozapine | P08588 | ADRB1 | 0.35261 |
| DB00349 | Clobazam | P23416 | GLRA2 | 0.35254 |
| DB00904 | Ondansetron | P08913 | ADRA2A | 0.35154 |
| DB00753 | Isoflurane | P21917 | DRD4 | 0.35093 |
| DB00315 | Zolmitriptan | P41595 | HTR2B | 0.35076 |
| DB00370 | Mirtazapine | P08588 | ADRB1 | 0.3504 |
| DB01242 | Clomipramine | P35368 | ADRA1B | 0.34992 |
| DB00494 | Entacapone | P28222 | HTR1B | 0.34934 |
| DB00246 | Ziprasidone | P29274 | ADORA2A | 0.34915 |
| DB00622 | Nicardipine | P25024 | CXCR1 | 0.34857 |
| DB00211 | Midodrine | P25024 | CXCR1 | 0.34854 |
| DB00246 | Ziprasidone | P18507 | GABRG2 | 0.34744 |
| DB01242 | Clomipramine | P25100 | ADRA1D | 0.34711 |
| DB01019 | Bethanechol | P20309 | CHRM3 | 0.34705 |
| DB01142 | Doxepin | P25024 | CXCR1 | 0.3468 |
| DB00408 | Loxapine | P20309 | CHRM3 | 0.34666 |
| DB00489 | Sotalol | P08913 | ADRA2A | 0.34622 |
| DB00674 | Galantamine | P23141 | CES1 | 0.34612 |
| DB00820 | Tadalafil | P35368 | ADRA1B | 0.34526 |
| DB00315 | Zolmitriptan | P35462 | DRD3 | 0.34511 |
| DB01019 | Bethanechol | P11229 | CHRM1 | 0.34481 |
| DB00408 | Loxapine | P11229 | CHRM1 | 0.34416 |
| DB00820 | Tadalafil | P25024 | CXCR1 | 0.343 |
| DB00496 | Darifenacin | P59768 | GNG2 | 0.34289 |
| DB00315 | Zolmitriptan | P18089 | ADRA2B | 0.34284 |
| DB00457 | Prazosin | P25024 | CXCR1 | 0.34274 |
| DB00820 | Tadalafil | P25100 | ADRA1D | 0.34274 |
| DB00477 | Chlorpromazine | Q01959 | SLC6A3 | 0.33853 |
| DB00312 | Pentobarbital | P21917 | DRD4 | 0.33845 |
| DB00408 | Loxapine | P08912 | CHRM5 | 0.33767 |
| DB01205 | Flumazenil | P21917 | DRD4 | 0.33765 |
| DB00186 | Lorazepam | P21917 | DRD4 | 0.33715 |
| DB00672 | Chlorpropamide | P23219 | PTGS1 | 0.33703 |
| DB00918 | Almotriptan | P08908 | HTR1A | 0.33658 |
| DB00843 | Donepezil | P02708 | CHRNA1 | 0.33648 |
| DB01174 | Phenobarbital | P21917 | DRD4 | 0.33606 |
| DB00654 | Latanoprost | P43115 | PTGER3 | 0.33563 |
| DB00370 | Mirtazapine | P28221 | HTR1D | 0.3355 |
| DB01036 | Tolterodine | P59768 | GNG2 | 0.3331 |
